# Supplementary material for: A Customizable Suite of Methods to Sequence and Annotate Cattle Antibodies
Source: Vaccines (Basel). 2023 Jun 14;11(6):1099. doi: 10.3390/vaccines11061099 (PMC10302312; doi:10.3390/vaccines11061099)
Supplement: Supplementary file 1 [file vaccines-11-01099-s001.zip › Supplementary Figures.pdf]

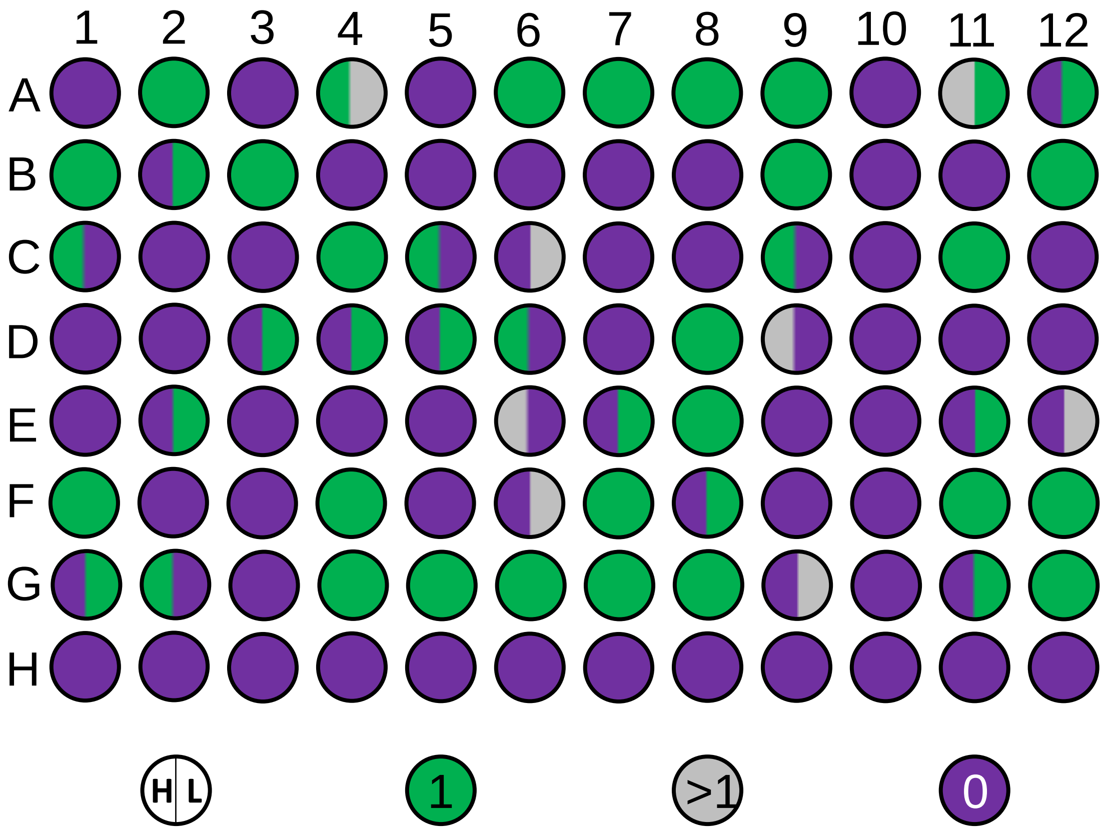

Figure S1A: Graphic representation of H and L chains recovery by the low throughput amplification and sequencing strategy from 84 single cells sorted in rows A to G. The left side of the well represents the H chain status and the right side represents the L chain status. Green represents a unique chain amplification, grey represents multiple chains amplified, and purple represents failed sequencing of a productive chain.

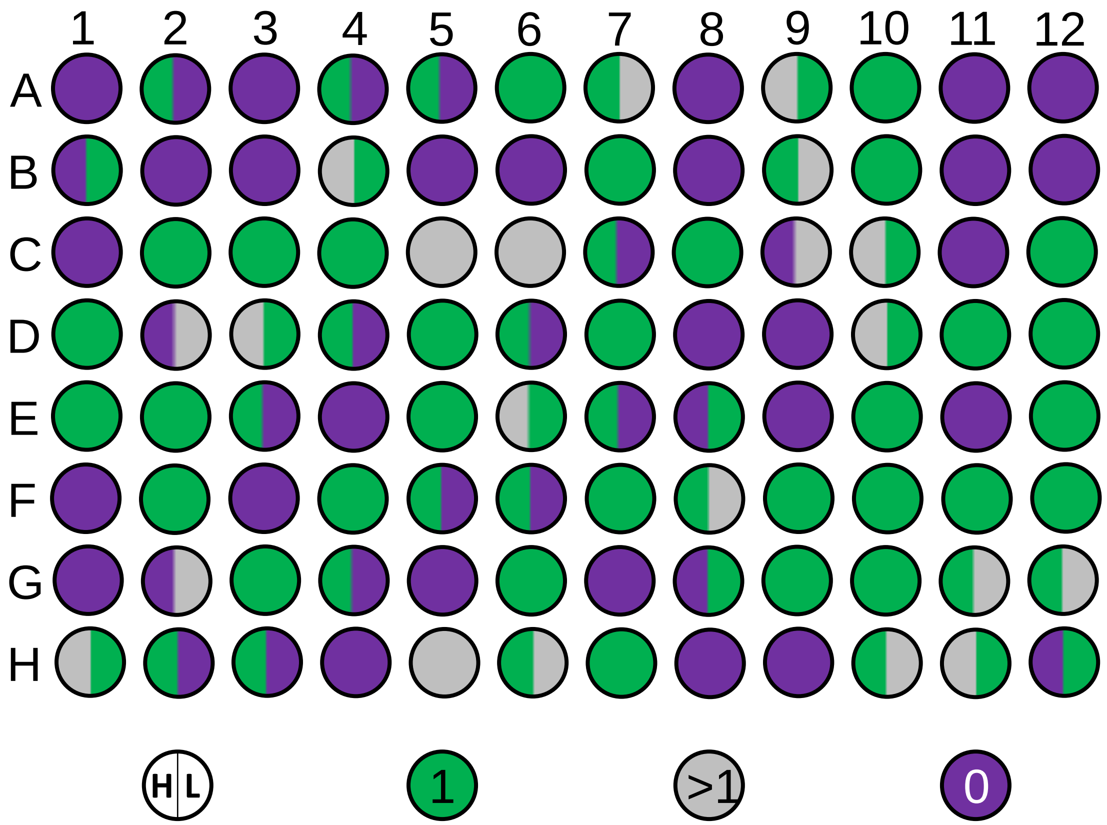

Figure S1B: Graphic representation of H and L chains recovery by the medium throughput amplification and sequencing strategy from 96 single cells sorted in rows A to H. The left side of the well represents the H chain status and the right side represents the L chain status. Green represents a unique chain amplification, grey represents multiple chains amplified, and purple represents failed sequencing of a productive chain.

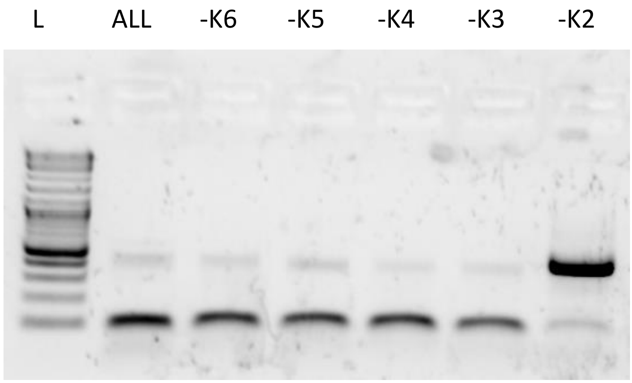

Figure S2: Testing IgK multiplexing. cDNA amplification of IgK rearrangements with IgK_C2 paired with all IgK V gene segment specific primers (ALL, amplicon expected size: ~500bp), or all except one of the forward primers at a time (-K2 to -K6 for IgK_L2 to IgK-L6 primers), showing that IgK_L1 inhibits the multiplexed reaction and cannot be included for multiplexing. L: NEB 1Kb Plus ladder.

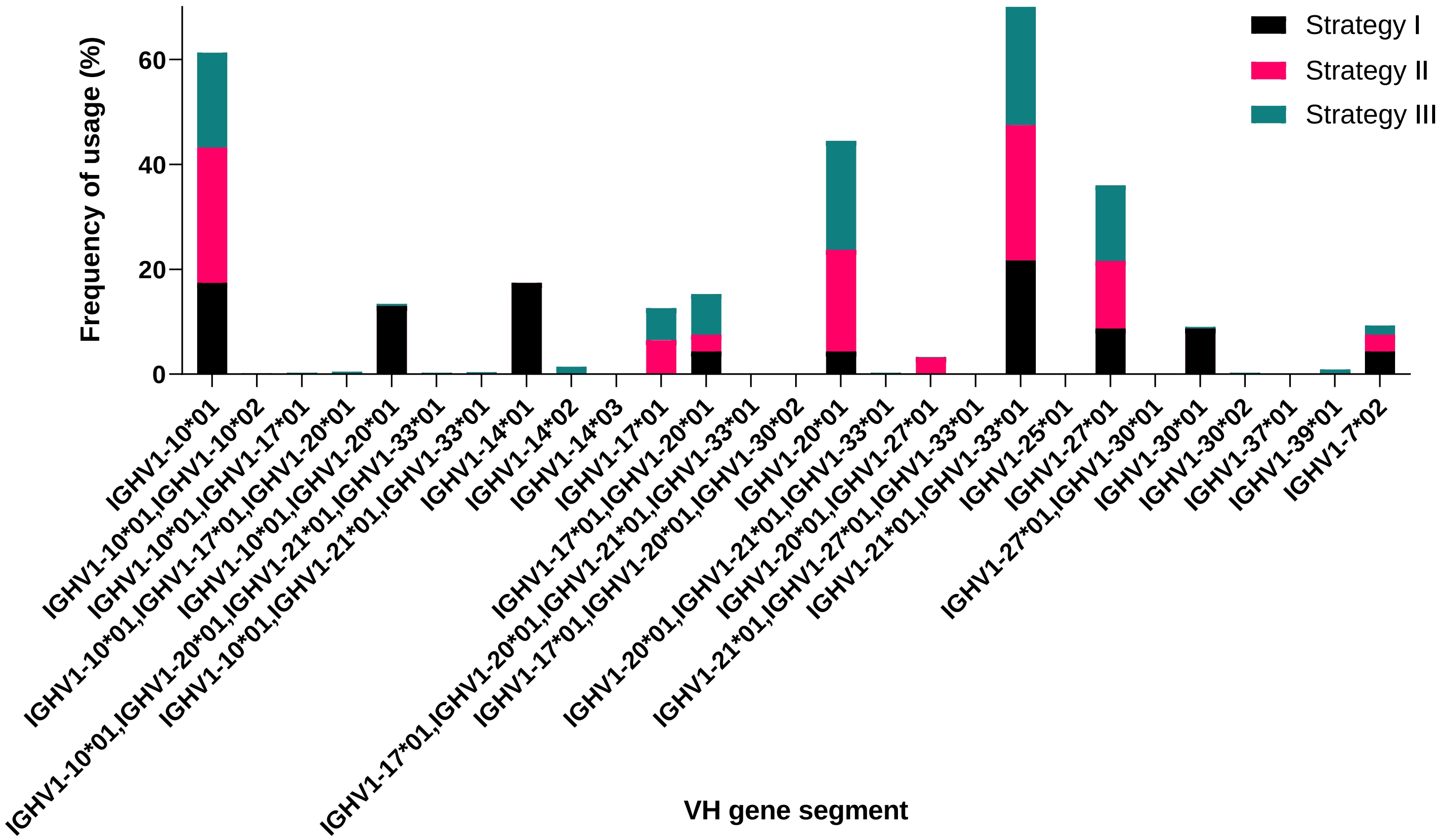

Figure S3: V_H gene segment usage. Usage frequencies of the heavy chain variable (V_H) gene segments used by antibody pairs recovered using amplification strategy I (black), strategy II (pink) and strategy III (cyan).
